# Supplementary material for: Identification of Secondary Biomechanical Abnormalities in the Lower Limb Joints after Chronic Transtibial Amputation: A Proof-of-Concept Study Using SPM1D Analysis
Source: Bioengineering (Basel). 2022 Jun 30;9(7):293. doi: 10.3390/bioengineering9070293 (PMC9311753; doi:10.3390/bioengineering9070293)
Supplement: Supplementary file 1 [file bioengineering-09-00293-s001.zip › bioengineering-1636384-supplementary.pdf]

bioengineering

MDPI

Identification of secondary biomechanical abnormalities in the lower limb joints after chronic transtibial amputation: A proof of concept study using SPM1D analysis

Amr Alhossary [1], Wei Tech Ang [1], Karen Chua Sui Geok [2], Matthew Rong Jie Tay [2], Poo Lee Ong [2], Tsurayuki Murakami [2], Tabitha Quake [2], Trevor Binedell [2], Seng Kwee Wee [2], Min Wee Phua [2], Yong Jia Wei [1] and Cyril John Donnelly [1,*]

[1] Rehabilitation Research Institute of Singapore, Nanyang Technological University, Singapore 308232, Singapore;
aalhossary@ntu.edu.sg (A.A.); wtang@ntu.edu.sg (W.T.A.); jiawei.yong@ntu.edu.sg (Y.J.W.)
[2] Centre of Rehabilitation Excellence, Tan Tock Seng Hospital, Singapore 308433, Singapore;
karen_chua@ttsh.com.sg (K.C.S.G.); matthew_rj_tay@ttsh.com.sg (M.R.J.T.);
poolee_ong@ttsh.com.sg (P.L.O.); tsurayuki_murakami@ttsh.com.sg (T.M.);
tabitha_zh_quake@ttsh.com.sg (T.Q.); trevor_binedell@ttsh.com.sg (T.B.);
seng_kwee_wee@ttsh.com.sg (S.K.W.); min_wee_phua@ttsh.com.sg (M.W.P.)
* Correspondence: cyril.donnelly@ntu.edu.sg; Tel.: +65-6904-1363

Demographic Data

Table S1. Group baseline demographic, clinical and locomotor characteristics of sample (n=12).

Baseline Characteristics	Value
Age (years)[a]	46.8 ± 14.6
Range	(26 - 73)
Gender n (%)	
- Male	12 (100%)
Race n (%)	
- Chinese	6 (50%)
- Malay	3 (25%)
- Indian	1 (8.3%)
- Eurasian	1 (8.3%)
- Caucasian	1 (8.3%)
Duration post event at study enrolment, days[a]	2982 ± 2955.2
Range	(906 - 9820)
Diagnoses	
- Transtibial amputation (below knee)	12 (100%)
Side of amputation	
- Right	8 (66.7%)
- Left	4 (33.3%)
Nature	
- Traumatic	9 (75%)
- Peripheral vascular disease (Diabetes Mellitus)	2 (16.7%)
- Others (congenital absence)	1 (8.33%)
Gait Speed (m/s)[a,b]	1.43 ± 0.26
Range	(1 – 1.85)
Distance walked (m)[a,c]	394.3 ± 57.8
Range	(312 – 500)
AMPPRO[d] /47[a]	45.5 ± 1.24
Range	(42 – 46)
K level[e]	
- K3	1 (8.33%)
- K4	11 (91.7%)
Walking aid n (%)	
- No	12 (100%)

Legend:.
[a]values presented as Mean ± SD.
[b]Determined using the 10-metre walk test (10 MWT).
[c]Determined using the 6-minute walk test (6 MWT).
[d]Determined using the Amputee Mobility Predictor with prosthesis (AMPPRO).
[e]K level: lower limb extremity prosthesis Medicare Functional Classification Levels.

Table S2. Individual baseline demographic, clinical and locomotor characteristics of sample (n=12).

Subject	Age	Gender	Race	Gait Speed[a] (m/s)	Distance walked[b] (m)	AMPPRO[c]/47	K Level[d] (K0-4)	Side	Nature	Duration post amputation (days)
**1**	33	M	Malay	1.67	500	46	K4	R	Traumatic	1772
**2**	73	M	Chinese	1.0	369	44	K4	R	Peripheral vascular disease (DM)[e]	Not available
**3**	52	M	Malay	1.28	417	46	K4	R	Traumatic	1034
**4**	45	M	Caucasian	1.75	415	46	K4	L	Traumatic	2292
**5**	52	M	Eurasian	1.70	450	46	K4	L	Others	Not available
**6**	26	M	Chinese	1.85	410	46	K4	R	Traumatic	906
**7**	55	M	Malay	1.21	353	46	K4	R	Traumatic	1472
**8**	53	M	Chinese	1.51	335	46	K4	R	Traumatic	9820
**9**	28	M	Chinese	1.27	436	46	K4	L	Traumatic	2314
**10**	35	M	Indian	1.40	420	46	K4	R	Traumatic	Not available
**11**	66	M	Chinese	1.32	315	46	K4	R	Traumatic	4247
**12**	44	M	Chinese	1.21	312	42	K3	L	Peripheral vascular disease (DM)[e]	Not available

Legend:.
[a] measured by 10 MWT :10-metre walk test.
[b] measured by 6MWT: 6-minute walk test.
[c] AMPPRO: Amputee Mobility Predictor.
[d] K level: lower limb extremity prosthesis Medicare Functional Classification Levels.
[e] DM: Diabetes Mellitus.
R: Right.
L : left.

Further plots of TTA patients versus control ref persons' recordings

In Supplementary Figure S1 and Supplementary Figure S2, we show the respective figures of main manuscript Figure 2 and Figure 3 after excluding the outlier person (# 6).

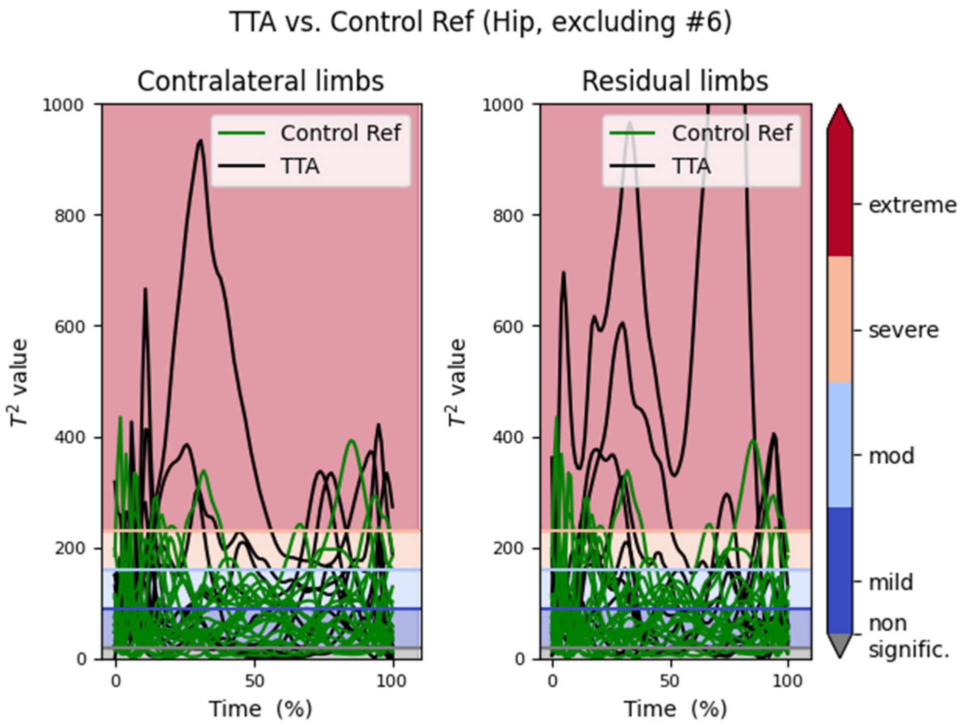

Figure S1. Transtibial Amputation patients recording vs Reference recordings of the hip joint, excluding patient # 6.

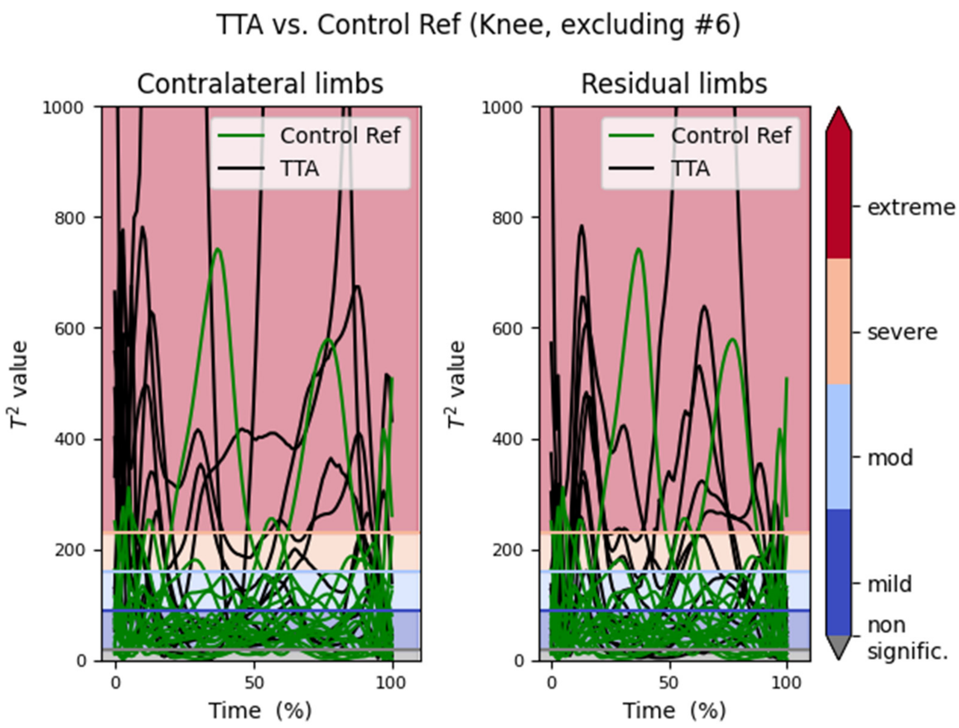

Figure S2. Transtibial Amputation patients recording vs Reference recordings of the knee joint, excluding patient # 6.

Differential analysis of deviations

To further analyze the results, we calculated the frequency of deviation per joint in our sample. In the following figures, we draw the histogram of deviation of the each TTA patients' joints. It is not logical to count the cases with very short and transient spikes as a case of severe deviation. Therefore, we considered 20% of the stance time to be the time threshold to consider a joint to be deviated. That is to say, if a joint has extreme deviation from the reference (shown as Red) for 25% of the time, it would be considered belonging to and will be counted with the category "Extreme". However, a joint that has extreme deviation (Red color) for only 5% of the time, severe deviation (Orange color) for 10% of the time and moderate deviation (Cyan color) for 10% of the time will be considered only moderate, because 20% of the time at least showed moderate deviation.

Looking to the contralateral limb in Supplementary Figure S3, the patients have either moderate (6 patients: 50%) or extreme (6 patients: 50%) deviations from the normative data set. All extreme deviations affect the contralateral knee, and they may or may not include other contralateral joints: 2 cases with isolated knee severe deviation, 2 cases with both Hip and knee and 2 cases with both knee and ankle.

However, in contrast, the situation in the residual limb (Supplementary Figure S4) showed that: 5 patients had extreme deviation in at least one joint. All of the 5 patients had extreme deviation in the hip and 3 thereof show extreme deviation in the knee as well.

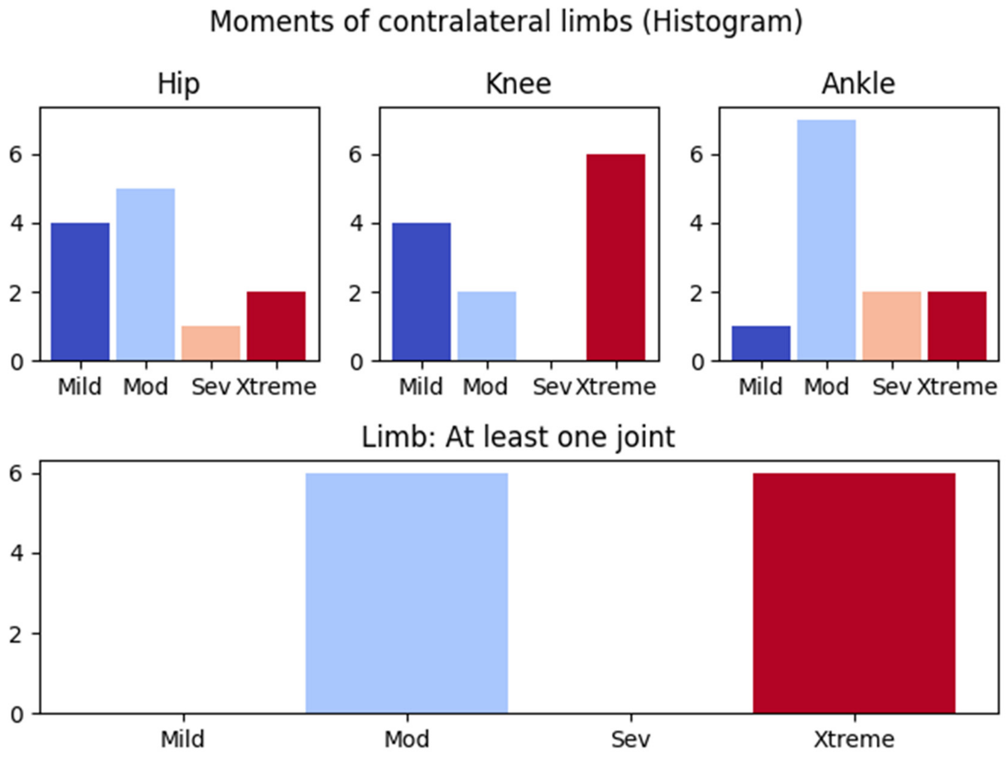

Figure S3. Histogram of deviation in TTA patients' contralateral limbs.

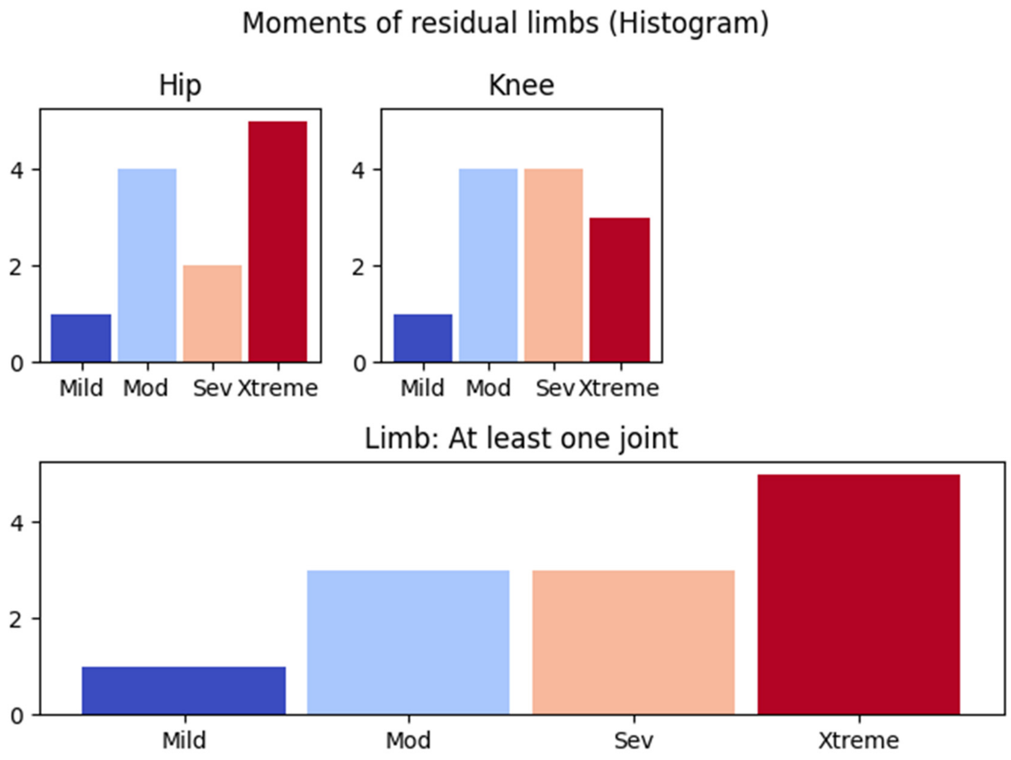

Figure S4. Histogram of deviation in TTA patients' residual limbs (no ankle joint).

Marker Placement

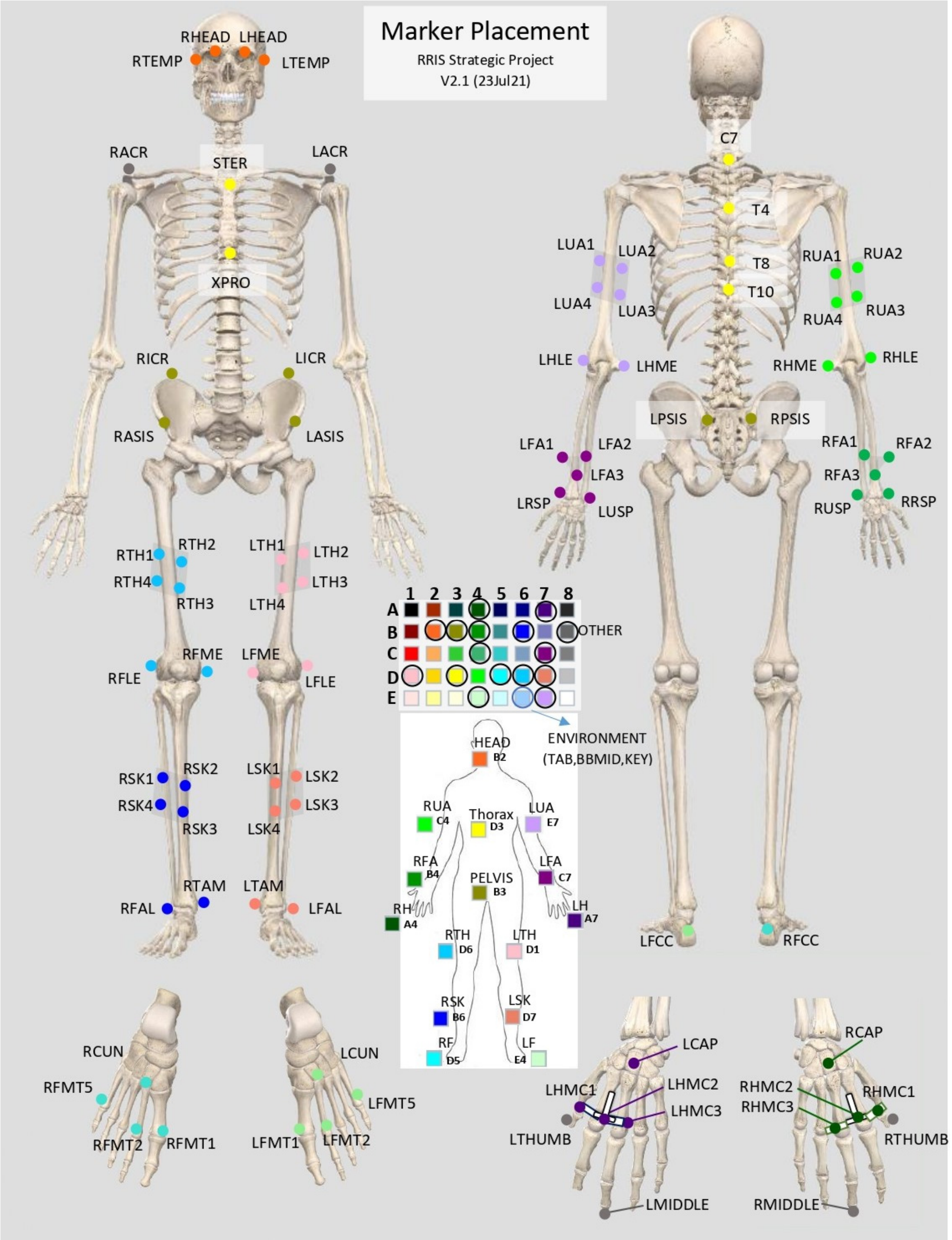

Figure S5. Marker Placement protocol.
